# *TBC1D4* ‐Related Monogenic Diabetes: Molecular Mechanisms, Clinical Recognition, and Therapeutic Implications

**DOI:** 10.1111/1753-0407.70266

**Published:** 2026-08-21

**Authors:** Wenjing Wang, Lidan Ma, Bingzi Dong, Peihan Gao, Xiaofang Sun

**Affiliations:** ^1^ Department of Endocrinology and Metabolic Diseases The Affiliated Hospital of Qingdao University Qingdao Shandong China

**Keywords:** GLUT4, insulin resistance, molecular genetics, monogenic diabetes, *TBC1D4* mutations

## Abstract

Monogenic diabetes caused by *TBC1D4* mutations is a relatively rare hereditary disorder of glucose metabolism that has increasingly gained attention in recent years. The protein encoded by this gene plays a pivotal regulatory role in glucose transport within skeletal muscle and adipose tissue; its dysfunction leads to significant insulin resistance and glucose dysregulation characterized by predominant postprandial hyperglycemia. Accumulating research indicates that *TBC1D4* mutations may contribute to metabolic disturbances by impairing glucose transport and blunting cellular insulin sensitivity. Patients typically present in adolescence or young adulthood, often accompanied by metabolic comorbidities such as obesity, dyslipidemia, fatty liver, and hyperuricemia; some may also manifest acanthosis nigricans or polyendocrine metabolic ovarian syndrome (PMOS; formerly polycystic ovary syndrome)‐like features. Due to frequent clinical misdiagnosis or underdiagnosis, a comprehensive evaluation incorporating age of onset, family history, glycemic characteristics, autoantibody status, and genetic testing is essential for accurate diagnosis. Currently, specific targeted therapies are lacking, and lifestyle intervention remains the cornerstone of management, with exercise intervention being particularly vital for improving insulin resistance. This review focuses on the molecular biological characteristics of the *TBC1D4* gene and its encoded protein, mutation types, pathogenic mechanisms, clinical manifestations, and diagnostic strategies, as well as therapeutic advances. It aims to systematically summarize the research progress on *TBC1D4*‐related monogenic diabetes to enhance clinical awareness and provide a reference for early identification, precise diagnosis, and individualized treatment.

## Highlights

*TBC1D4*‐related monogenic diabetes is a rare insulin resistance syndrome characterized by prominent postprandial hyperglycemia and frequent misdiagnosis. Dysfunction of TBC1D4/AS160 impairs GLUT4 trafficking in skeletal muscle, linking genotype to disturbed glucose metabolism and heterogeneous clinical phenotypes. Early recognition supported by genetic testing, together with exercise‐centered lifestyle intervention, individualized nutritional management, and appropriate pharmacotherapy, may improve diagnosis and long‐term management.

## Graphical abstract

TBC1D4‐related monogenic diabetes is characterized by defective AS160‐mediated GLUT4 trafficking, skeletal muscle insulin resistance, and predominant postprandial hyperglycemia. Clinical recognition relies on atypical young‐onset diabetes with marked insulin resistance, followed by genetic confirmation and individualized management.

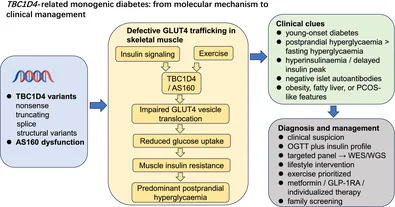

## 1 Introduction

Monogenic diabetes represents a heterogeneous group of hyperglycemic disorders caused by a single pathogenic gene variant. It is primarily characterized by impaired insulin secretion due to defects in pancreatic β‐cell function or development [1], whereas obesity and insulin resistance are generally not predominant features. To date, more than 40 subtypes involving over 40 distinct genes have been identified. Their inheritance patterns encompass autosomal dominant (the most common form, such as mutations in *GCK* or *HNF1A*) and autosomal recessive modes [2]. Its classification encompasses monogenic insulin resistance syndromes, neonatal diabetes mellitus (NDM, occurring within the first 6 months of life with an incidence of approximately 1/90 000), and maturity‐onset diabetes of the young (MODY, typically diagnosed before age 25 and accounting for 0.5%–5% of nonautoimmune diabetes cases). Additionally, it includes various diabetes‐associated syndromes such as Wolfram syndrome and Wolcott‐Rallison syndrome [2]. Monogenic diabetes transcends the traditional classifications of diabetes. As neither an autoimmune disease nor a typical insulin‐resistant Type 2 diabetes, it constitutes a continuous clinical spectrum that exhibits phenotypic overlap with both Type 1 and Type 2 diabetes. Consequently, this leads to frequent underdiagnosis or misdiagnosis [3]. Identifying these cases is of paramount importance, as they possess unique therapeutic implications.

In 2009, a research team from the University of Cambridge and colleagues first reported that a truncated mutation in the *TBC1D4* gene, R363X (c.1087C>T), causes insulin resistance in humans. This was initially identified in a pedigree presenting with acanthosis nigricans and postprandial hyperinsulinemia [4]. Subsequently, in 2014, Moltke et al. published a landmark study in *Nature*, reporting that a nonsense mutation in *TBC1D4*, R684X (p.Arg684Ter), is significantly associated with Type 2 diabetes susceptibility in the Greenlandic population [5]. This discovery highlighted *TBC1D4* as a critical causative gene for glucose dysregulation and established its pivotal role in the study of monogenic insulin resistance‐related diabetes. This review systematically summarizes the molecular genetic basis, pathogenic mechanisms, and clinical diagnosis and management of monogenic diabetes caused by *TBC1D4* mutations.

## 2 *TBC1D4* Gene and AS160 Biology

### 2.1 *TBC1D4* Structure and Function

The human TBC1D4 gene is located on chromosome 13q22.2 and comprises approximately 23 exons and 22 introns [6]. It encodes a protein with a relative molecular mass of approximately 160 kDa. This protein was originally identified by Kane and colleagues as an insulin‐regulated substrate of Akt, leading to its designation as AS160 (Akt substrate of 160 kDa) [7, 8]. As a member of the TBC domain‐containing protein family, TBC1D4 exhibits tissue‐specific isoform expression: its short isoforms are ubiquitously expressed, whereas the long isoforms are predominantly expressed in skeletal muscle and are also functionally expressed in the heart [9, 10]. These tissue‐specific isoforms play important roles in maintaining systemic glucose homeostasis [11]. As a key downstream effector of the insulin signaling pathway, TBC1D4/AS160 primarily mediates the intracellular translocation and membrane fusion of glucose transporter 4 (GLUT4) vesicles upon insulin stimulation, thereby regulating glucose uptake in peripheral tissues [11, 12, 13]. Recent studies have demonstrated that TBC1D4 serves as a central molecular determinant of insulin sensitivity in skeletal muscle. Moreover, its intimate involvement in postprandial glucose metabolism, energy homeostasis, and heritable insulin‐resistant phenotypes positions it as a critical entry point for elucidating the transition from molecular signaling aberrations to metabolic disorders [14].

### 2.2 AS160 Structure and Domains

The encoded AS160 is a multidomain functional protein characterized by several critical regions. It comprises two N‐terminal phosphotyrosine‐binding (PTB) domains, a calmodulin‐binding domain (CBD), multiple Akt‐ and AMPK‐dependent phosphorylation sites, and a C‐terminal TBC (Tre‐2/Bub2/Cdc16) domain [15]. Although the PTB domains have been less extensively studied than the TBC domain, accumulating evidence indicates that they play important regulatory roles. In particular, the second PTB domain mediates interaction with insulin‐regulated aminopeptidase (IRAP), thereby contributing to GLUT4 vesicle localization, while PTB‐mediated phospholipid binding facilitates membrane recruitment of AS160 [12, 16, 17]. Furthermore, genetic studies of the homologous gene *TBC1D1* suggest that alterations affecting PTB domains may contribute to metabolic dysfunction, highlighting the potential physiological importance of these domains [17, 18]. The C‐terminal TBC domain possesses Rab GTPase‐activating protein (Rab‐GAP) activity that promotes the conversion of Rab proteins from the active GTP‐bound state to the inactive GDP‐bound state [7, 11]. Multiple Akt‐ and AMPK‐dependent phosphorylation sites undergo dynamic modification in response to insulin or exercise stimuli, thereby regulating protein activity and its interactions with downstream effectors [12, 13, 14]. Together, these structural domains underpin the diverse regulatory functions of AS160.

### 2.3 AS160 in Insulin Signaling

**FIGURE 1:**
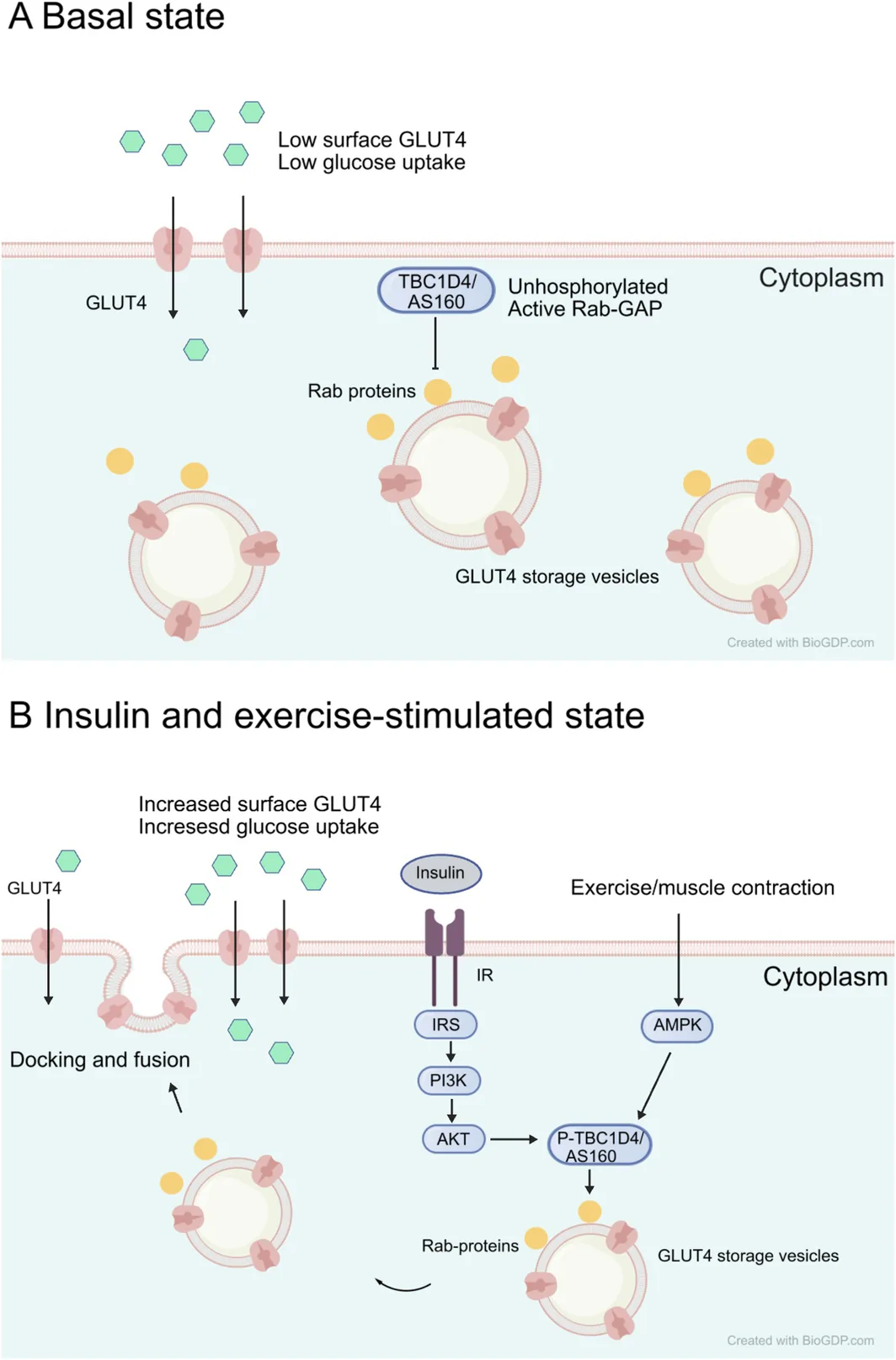
Schematic illustration of TBC1D4/AS160‐mediated regulation of GLUT4 translocation in skeletal muscle. (A) In the basal state, unphosphorylated TBC1D4/AS160 exhibits Rab‐GAP activity, leading to intracellular retention of GLUT4 storage vesicles (GSVs), reduced surface GLUT4 abundance, and low glucose uptake. (B) Insulin induces TBC1D4/AS160 phosphorylation through the IR–IRS–PI3K–Akt pathway, whereas exercise/muscle contraction acts through the AMPK pathway, thereby promoting GLUT4 storage vesicle translocation, docking, and membrane fusion, and increasing glucose uptake in skeletal muscle. This figure was summarized from the literature and created with BioGDP [19].

AS160 serves as a pivotal hub within the insulin signaling pathway and plays a decisive role in maintaining glucose homeostasis [11, 12, 13]. Research indicates that in the basal state, in the absence of insulin stimulation, the TBC domain “locks” GLUT4‐containing vesicles intracellularly by acting on target Rab proteins (such as Rab2A, Rab8A, Rab10, and Rab14), thereby inhibiting glucose uptake(Figure 1A) [7]. Upon insulin stimulation, the insulin receptor activates Akt via the IRS–PI3K–Akt signaling axis. Activated Akt subsequently phosphorylates key residues of AS160, leading to the inhibition of its Rab‐GAP activity. This suppression facilitates the translocation and membrane fusion of GLUT4 storage vesicles (GSVs) from the intracellular compartment to the plasma membrane, thereby increasing the density of surface GLUT4 and ultimately enhancing glucose uptake in skeletal muscle and adipose tissues(Figure 1B) [12]. In other words, AS160 acts as a critical “bridge” between insulin signaling and GLUT4 translocation, serving as a key effector molecule that determines insulin responsiveness in peripheral tissues [13].

Beyond the classical insulin signaling pathway, exercise and muscle contraction can also regulate AS160 phosphorylation through pathways such as AMPK [11]. Exercise‐induced phosphorylation of AS160 promotes GLUT4 translocation independently of insulin and contributes to enhanced skeletal muscle insulin sensitivity during the postexercise period. Persistent AS160 phosphorylation is thought to facilitate subsequent insulin‐stimulated GLUT4 trafficking, thereby improving glucose disposal following exercise [14, 20, 21]. These findings further underscore the central role of AS160 in coordinating insulin‐ and exercise‐mediated regulation of skeletal muscle glucose uptake. Beyond these glucose‐centered effects, emerging evidence suggests that AS160, together with the closely related RabGAP TBC1D1, also participates in the broader regulation of skeletal muscle substrate metabolism. These RabGAPs have been implicated in long‐chain fatty acid uptake [22], whereas combined deficiency of *Tbc1d1* and *Tbc1d4* in mice shifts substrate utilization toward lipid oxidation, supporting their roles in substrate preference and metabolic flexibility [23].

## 3 *TBC1D4* Genetics and Pathogenesis

### 3.1 Reported *TBC1D4* Mutations

To date, only eight pathogenic variants of *TBC1D4* have been reported in the global literature (Table 1). These are predominantly truncating variants, including nonsense mutations, frameshift mutations, and splicing aberrations that lead to premature protein termination. Representative examples include the R363X truncation mutation initially identified in early pedigree studies and the p.Arg684Ter (R684X) variant, which occurs at a relatively high frequency in the Greenlandic population [4, 5]. In 2009, Dash et al. screened the coding regions of *TBC1D4* in 156 patients with severe insulin resistance [24]. The R363X truncation mutation was first identified in a pedigree characterized by acanthosis nigricans and extreme postprandial hyperinsulinemia, suggesting that *TBC1D4* truncating variants contribute directly to the manifestation of heritable insulin‐resistant phenotypes [4]. The 2014 study of the Greenlandic population further confirmed that p.Arg684Ter is not merely a sporadic case but a highly prevalent pathogenic variant in specific populations. It is significantly associated with skeletal muscle insulin resistance, postprandial hyperglycemia, and a markedly increased risk of Type 2 diabetes, thus representing a major milestone in *TBC1D4* research [5]. In recent years, with the advancement of whole‐exome sequencing (WES), whole‐genome sequencing (WGS), and structural variation (SV) analysis, novel pathogenic forms such as complex genomic rearrangements have also been identified [25], suggesting that the mutation spectrum of *TBC1D4* is continuously expanding. In addition, emerging case studies have identified previously unreported rare variants in diverse populations worldwide, further supporting the marked genetic heterogeneity of TBC1D4 [25, 26].

**TABLE 1:** Summary of reported TBC1D4 gene mutations worldwide.

Case/study	Variant/location	Mutation type
2006 [18]	R125W	Missense
2009 [4]	R363X	Nonsense (truncation)
2009 [24]	N1206S	Missense
2009 [24]	N655Y	Missense
2009 [24]	N785K	Missense
2014 [5]	R684X (p.Arg684Ter)	Nonsense
2024 [25]	DUP‐TRP/INV‐DUP	Complex rearrangement
2025 [26]	c.2199‐113A>G	Nonsense

Abbreviations: DUP‐TRP, duplication‐triplication; INV‐DUP, inverted duplication; WES, whole‐exome sequencing.

### 3.2 Mechanisms of Loss of Function

Current evidence suggests that the central pathogenic mechanism of *TBC1D4* mutations involves impaired regulation of GLUT4 vesicle trafficking resulting from loss of protein function [27]. Early functional studies on the R363X mutation revealed that this truncation occurs upstream of the GAP domain, resulting in the complete loss of the entire GAP domain and all subsequent downstream functional regions. This deletion abolishes the Rab‐GAP activity of the mutant protein. TBC1D4 regulates multiple Rab GTPases, including Rab2A, Rab8A, Rab10, Rab14, and Rab28 [7, 28, 29, 30]. Although Rab14 has been implicated in GLUT4 vesicle trafficking, current evidence suggests that it is one of several physiological substrates of TBC1D4 rather than the exclusive downstream target. Accordingly, loss of Rab‐GAP activity is expected to result in prolonged activation of multiple downstream Rab GTPases, thereby disrupting coordinated intracellular GLUT4 vesicle trafficking [4, 28, 30]. Furthermore, the pathogenic mechanisms of *TBC1D4* involve functional impairment of critical Akt phosphorylation sites. This disruption compromises the protein's ability to undergo normal inactivation in response to insulin signaling, thereby preventing the efficient translocation of GLUT4 storage vesicles from the intracellular compartment to the plasma membrane [31]. Recent studies of human skeletal muscle harboring the p.Arg684Ter variant further revealed that this mutation leads to a marked downregulation or absence of the long *TBC1D4* isoform, accompanied by reduced GLUT4 protein levels. These findings suggest that certain mutations may contribute to pathogenesis by compromising protein stability, altering the expression of tissue‐specific isoforms, and diminishing the levels of downstream glucose transporters [14]. Moreover, the phenotypic severity in some patients cannot be fully explained by a single *TBC1D4* variant alone. Consequently, some researchers have proposed a “second‐hit” hypothesis, suggesting that genetic defects may synergize with factors such as obesity, pubertal hormonal shifts, modifier genes, or metabolic stress to determine disease penetrance and clinical severity [1, 4]. However, this mechanism remains largely speculative and currently lacks robust, direct experimental evidence.

### 3.3 Genotype–Phenotype Analysis

Current genotype–phenotype analyses suggest that different types of *TBC1D4* variants (e.g., truncating versus missense variants) and distinct dosage effects (heterozygous versus homozygous states) are associated with different clinical presentations. Overall, truncating variants and biallelic mutations are generally linked to more profound loss of protein function and more severe clinical phenotypes [5, 25]. In contrast, variants retaining partial residual function may present only with mild glucose intolerance or later‐onset metabolic abnormalities [5]. Population studies have demonstrated a clear dosage effect for the p.Arg684Ter variant in Greenlanders: homozygous carriers exhibit significantly more severe skeletal muscle insulin resistance, elevated 2‐h glucose levels, and higher diabetes risk compared to heterozygous carriers [5]. Furthermore, *TBC1D4*‐related phenotypes do not follow a simple one‐to‐one correlation; rather, obesity, puberty, physical activity levels, and other genetic factors may act as modifiers. For instance, subsequent research suggests that physical activity can mitigate postprandial hyperglycemia in p.Arg684Ter homozygotes to some extent, indicating that environmental factors can partially buffer the metabolic aberrations caused by genetic defects [32]. In summary, *TBC1D4*‐related diabetes is characterized by significant genetic and clinical heterogeneity.

## 4 Clinical Features and Diagnosis

### 4.1 Clinical Features

*TBC1D4* mutation‐related diabetes is clinically rare, with a core phenotype characterized by profound peripheral insulin resistance that primarily affects skeletal muscle glucose uptake. Clinically, it frequently manifests as hyperinsulinemia, prominent postprandial hyperglycemia, and the coexistence of varying degrees of obesity and metabolic syndrome components [14, 27, 32]. Regarding the glycemic profile, the most suggestive characteristic is that postprandial hyperglycemia is more pronounced than fasting hyperglycemia. Oral glucose tolerance tests (OGTT) typically reveal a significant elevation in 2‐h glucose levels, often accompanied by a delayed or abnormally exaggerated insulin secretion peak. This pattern is consistent with impaired postprandial glucose clearance primarily driven by skeletal muscle defects [5, 14, 24]. At the same time, a subset of patients with *TBC1D4* mutations frequently presents with metabolic syndrome components, including obesity, dyslipidemia, metabolic dysfunction‐associated steatotic liver disease (MASLD) [33], hypertension, and hyperuricemia. Recently, the term polycystic ovary syndrome (PCOS) has been renamed polyendocrine metabolic ovarian syndrome (PMOS) through an international consensus process to better reflect its systemic endocrine and metabolic nature. Accordingly, the terminology PMOS (formerly PCOS) is adopted throughout this review. In some female patients, hyperandrogenism or PMOS (formerly PCOS)‐like manifestations have also been observed. These findings indicate that *TBC1D4* deficiency is not limited to glycemic dysregulation but encompasses a broad spectrum of metabolic disturbances [26, 27, 34, 35, 36].

### 4.2 Clinical Diagnosis

From a diagnostic perspective, *TBC1D4*‐related monogenic diabetes should be considered in adolescents or young adults with an atypical Type 2 diabetes‐like phenotype accompanied by marked insulin resistance. Key clinical red flags prompting further genetic evaluation include: (1) marked postprandial hyperglycemia with relatively preserved fasting glucose; (2) hyperinsulinemia or a delayed insulin peak during an oral glucose tolerance test (OGTT); (3) negativity for pancreatic islet autoantibodies; (4) a family history suggestive of monogenic inheritance; and (5) coexisting obesity, MASLD, PMOS (formerly PCOS)‐like manifestations, or other indicators of severe insulin resistance [3, 26, 34, 37, 38].

**FIGURE 2:**
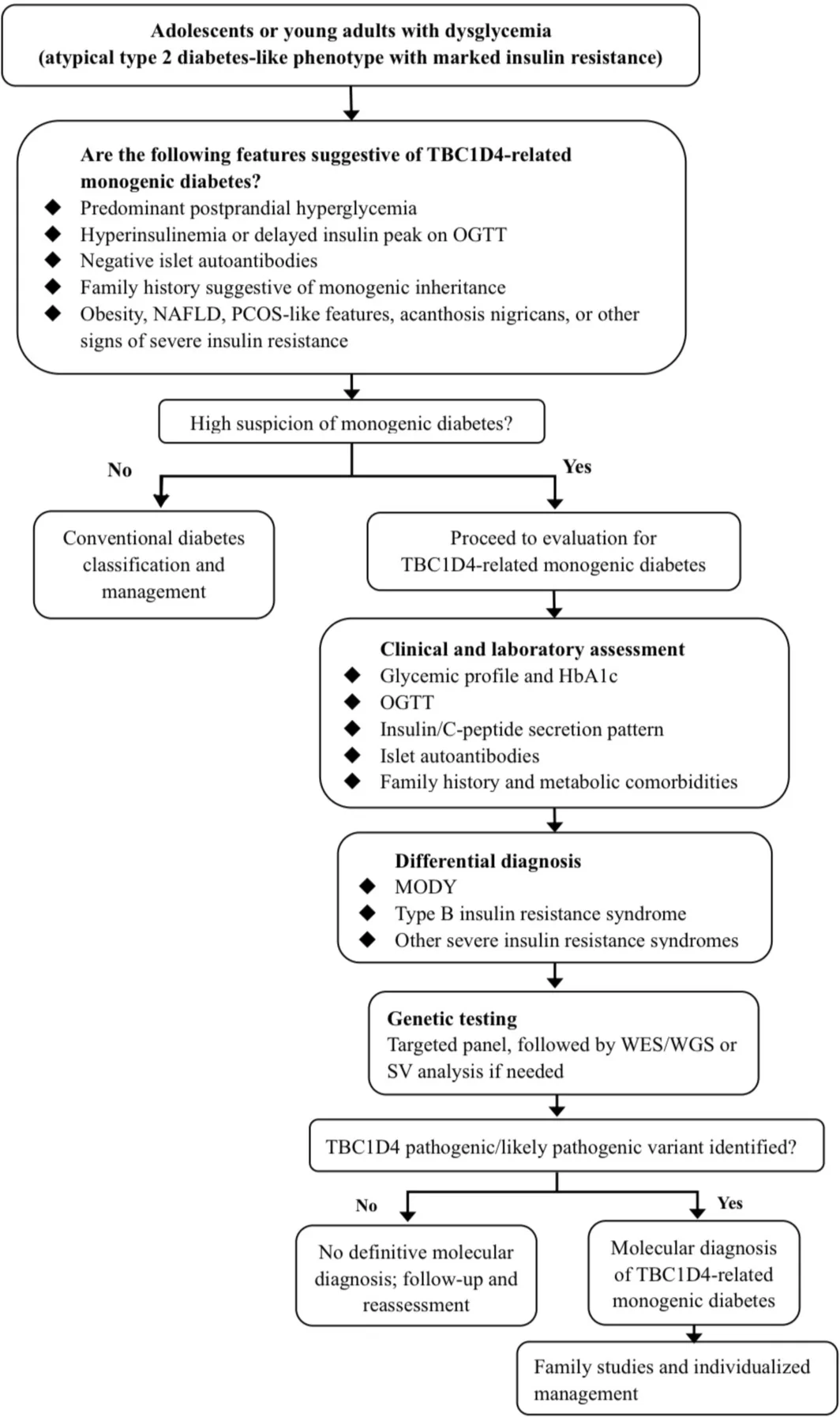
Clinical recognition and genetic testing workflow for TBC1D4‐related monogenic diabetes. This flowchart summarizes the clinical recognition, differential diagnosis, and stepwise genetic testing strategy for TBC1D4‐related monogenic diabetes. In adolescents or young adults with dysglycemia and an atypical type 2 diabetes‐like phenotype accompanied by marked insulin resistance, the presence of clinical red flags should prompt further evaluation and genetic testing. Identification of a pathogenic or likely pathogenic TBC1D4 variant supports the molecular diagnosis and guides family studies and individualized management.

Current diagnostic strategies for monogenic diabetes and monogenic insulin resistance syndromes generally emphasize the principle of “clinical screening followed by precise genetic confirmation.” The core of this approach involves stratifying and identifying suspected cases based on age of onset, family history, metabolic phenotypes, autoantibody status, and C‐peptide levels. Building on this clinical suspicion, targeted gene panels, WES, or, when necessary, WGS are employed to identify pathogenic variants and achieve a definitive diagnosis [3, 34, 37, 38]. Therefore, for *TBC1D4*‐related diabetes, patients with a high clinical suspicion but negative results from routine testing should be considered for deeper genomic investigation [11]. Its diagnosis should transcend the traditional boundaries of “diabetes subtyping” and be integrated into the broader framework of monogenic diabetes for identification and molecular confirmation. To facilitate clinical recognition and diagnostic stratification, the workflow for the recognition and genetic testing of TBC1D4‐related monogenic diabetes is summarized in Figure 2.

### 4.3 Differential Diagnosis

#### 4.3.1 MODY

Maturity‐Onset Diabetes of the Young (MODY) manifests before age 25 and is primarily caused by defects in pancreatic β‐cell function, with diagnosis confirmed through genetic testing for pathogenic variants in genes such as *GCK*, *HNF1A*, and *HNF4A* [39]. While characterized by young‐onset, family clustering, and preserved endogenous insulin secretion, most MODY subtypes do not feature prominent insulin resistance [38, 40]. In contrast, *TBC1D4*‐related disease is distinguished by hyperinsulinemia, marked postprandial hyperglycemia, and predominantly muscle‐driven peripheral insulin resistance.

#### 4.3.2 Type B Insulin Resistance

Type B insulin resistance syndrome is a rare autoimmune disorder caused by autoantibodies directed against the insulin receptor. It is more prevalent in females aged 0–60 years, with a female‐to‐male ratio of 2:1. Clinically, it is characterized by refractory hyperglycemia often requiring massive doses of insulin, acanthosis nigricans, and hyperandrogenism. Approximately one‐third of patients have coexisting autoimmune diseases, such as systemic lupus erythematosus (SLE). Compared to *TBC1D4*‐related diabetes, Type B syndrome typically presents with a more acute onset and greater clinical severity. Diagnosis relies on the detection of extreme hyperinsulinemia and insulin receptor autoantibodies [41, 42].

#### 4.3.3 Other Severe Insulin Resistance

Differential diagnosis is also required to distinguish *TBC1D4* variants from other severe insulin resistance (SIR) syndromes, such as Type A insulin resistance caused by *INSR* mutations, Rabson‐Mendenhall syndrome, lipatrophic diabetes, and other hereditary forms of extreme insulin resistance [1]. These conditions are often characterized by more pronounced dysmorphic features, extreme hyperinsulinemia, severe metabolic derangements, and abnormal fat distribution [27]. In contrast, *TBC1D4* mutations typically manifest as partial defects in insulin signaling, characterized primarily by postprandial hyperglycemia and skeletal muscle‐specific insulin resistance.

## 5 Therapeutic Advances

### 5.1 Lifestyle Interventions

Consistent with other forms of monogenic diabetes, there is currently no universally accepted targeted therapy for *TBC1D4*‐related diabetes. Management primarily aims to improve insulin sensitivity and prevent long‐term diabetic complications, with lifestyle intervention remaining the cornerstone of treatment and long‐term disease management. Exercise intervention holds particular importance, as skeletal muscle contraction can partially bypass the classical insulin receptor‐IRS‐PI3K‐Akt signaling defects. It promotes GLUT4 translocation to the plasma membrane via the AMPK‐TBC1D1/4‐GLUT4 pathway, thereby enhancing muscle glucose uptake and mitigating postprandial hyperglycemia [43, 44, 45]. Evidence from animal and skeletal muscle mechanistic studies indicates that AMPK activation can enhance post‐exercise insulin sensitivity through *TBC1D4*‐related pathways. This provides a clear molecular rationale for prioritizing intensive exercise intervention in patients with *TBC1D4* deficiency [45]. Population studies in Greenlanders estimate that, compared with a sedentary lifestyle, approximately 1 h of high‐intensity physical activity per day is associated with an additional reduction of 0.7 mmol/L in 2‐h OGTT glucose levels among homozygous carriers [32]. Consistent with these clinical observations, a recent study using *Tbc1d4*‐deficient mice demonstrated that chronic endurance exercise significantly improved glucose tolerance and insulin sensitivity, indicating that exercise can partially overcome genetically induced peripheral insulin resistance associated with TBC1D4 deficiency [46]. Together, these findings further support exercise as a particularly beneficial intervention for individuals with TBC1D4 deficiency. According to the 2022 Expert Consensus from the American College of Sports Medicine (ACSM), exercise intervention for patients with diabetes or insulin resistance should adopt a comprehensive regimen based on regular aerobic exercise combined with resistance training. Additionally, efforts should be made to minimize sedentary time in daily life [47]. For aerobic exercise, it is recommended to perform at least 150 min of moderate‐intensity activity (e.g., brisk walking, cycling, swimming) or 75 min of high‐intensity activity per week. These sessions should be distributed over at least 3 days, with no more than two consecutive days between sessions, to maintain glycemic control and reduce HbA1c. In addition, resistance training performed 2–3 times per week, including free weights, machines, resistance bands, or bodyweight exercises, may further enhance muscle mass and insulin sensitivity [47].

Regarding dietary management, current clinical guidelines generally recommend adherence to established nutritional principles for insulin resistance and metabolic syndrome. These include total energy restriction, reduced intake of fat and simple sugars, adequate consumption of high‐quality protein, regular meal patterns, and targeted weight management [37]. Particularly for patients presenting with obesity, MASLD, hyperuricemia, or PMOS (formerly PCOS)‐like manifestations, weight loss itself can reduce the overall insulin resistance burden and ameliorate postprandial hyperglycemia [1, 37]. Beyond these general recommendations, emerging evidence suggests that dietary responses in individuals with *TBC1D4*‐associated metabolic dysfunction may vary according to genetic background. A randomized crossover study in Greenlandic Inuit demonstrated that a traditional marine‐based diet modestly improved daily glycemic control compared with a Western diet, although its effects on glucose tolerance and insulin sensitivity appeared to differ according to *TBC1D4* genotype, suggesting a potential gene–diet interaction [48]. Together with broader evidence indicating that nutritional responses may be genotype dependent [49], these findings support further investigation of individualized dietary strategies for genetically influenced metabolic dysfunction.

### 5.2 Pharmacotherapy

In terms of pharmacological treatment, individualized combination therapies are clinically employed to improve insulin sensitivity, manage weight, and control hyperglycemia. Potential options include metformin, GLP‐1 receptor agonists (GLP‐1 RAs), and, if necessary, insulin. However, current evidence is predominantly derived from case reports or small‐scale observational studies, and a standardized therapeutic protocol specifically for *TBC1D4*‐related disease remains established [1, 37]. Based on the general management principles for severe insulin resistance (SIR) syndromes, metformin may serve as a foundational therapy, particularly for patients with overweight or obesity and prominent insulin resistance [1]. For individuals with significant weight gain or marked postprandial hyperglycemia, GLP‐1 RAs also hold substantial theoretical and clinical value [26]. Insulin secretagogues, such as sulfonylureas and meglitinides, are generally not prioritized as first‐line therapy for these patients because this disorder is mainly characterized by peripheral insulin resistance and postprandial dysglycemia; therefore, further stimulation of endogenous insulin secretion may not address the principal pathophysiological defect, while these agents may also increase the risks of hypoglycemia and weight gain [1, 4, 50, 51]. On the other hand, a 2024 case report of severe insulin‐resistant diabetes caused by a complex *TBC1D4* rearrangement suggested that some patients may exhibit limited responses to multiple therapeutic interventions. This underscores the significant heterogeneity in pharmacological sensitivity among individuals with this disorder [25].

### 5.3 Gene Therapy

Looking ahead, gene editing, cell‐based therapies, and small‐molecule agents targeting the Rab‐GTPase/TBC1D4 regulatory network may offer promising precision medicine strategies for this group of monogenic insulin resistance syndromes. Technically, while CRISPR‐Cas9, base editing, and other gene‐editing strategies have been extensively discussed as potential avenues for treating monogenic diabetes, they remain in the early stages of exploration and have not yet been translated into clinical practice [52, 53]. Furthermore, given that the core pathology of *TBC1D4* deficiency is intricately linked to Rab‐GTPase regulation, GLUT4 vesicle translocation, and impaired skeletal muscle glucose clearance, the development of small‐molecule drugs targeting the Rab‐GTPase/TBC1D4/GLUT4 signaling axis has a clear theoretical basis; however, current research remains largely confined to mechanistic exploration [45]. Overall, the treatment of *TBC1D4*‐related diabetes is undergoing a paradigm shift from traditional “glucose‐lowering control” toward “genotype‐guided precision metabolic management.” In the short term, the therapeutic focus remains on intensive lifestyle modifications combined with individualized pharmacological interventions.

## 6 Challenges and Future Perspectives

At present, research on *TBC1D4*‐related diabetes remains predominantly in the stage of case reports and small‐scale studies. Existing evidence is largely derived from early familial reports, studies on high‐frequency variants in specific populations, and recent sporadic cases. Although these investigations have continuously expanded the clinical spectrum of *TBC1D4*‐related disorders, the overall sample size remains limited and the study populations exhibit significant heterogeneity, which is insufficient to establish a robust clinical classification framework. Secondly, functional evidence remains limited. Although existing studies have shown that *TBC1D4* deficiency is associated with severe skeletal muscle insulin resistance, downregulation of GLUT4‐related proteins, and impaired postprandial glucose clearance, these findings have largely been derived from a small number of representative variants, particularly p.Arg684Ter [14]. Large‐scale and standardized functional validation studies across diverse variant types are still lacking. In addition, evidence supporting precision treatment for monogenic diabetes remains weak overall, and substantial gaps must still be addressed before true evidence‐based precision therapy can be achieved in this field [37, 54].

In the future, multicenter collaboration will be needed to further define the clinical spectrum of *TBC1D4*‐related disease. Moreover, the establishment of international patient registries and long‐term follow‐up systems will be essential for improving understanding of the natural history and long‐term outcomes of this condition [55, 56]. Established models in the field of monogenic diabetes provide mature experience for such endeavors. For instance, the University of Chicago Monogenic Diabetes Registry has accumulated a large number of families and confirmed cases, providing an empirical foundation for natural history studies, diagnostic optimization, and cascade screening within families [57]. Furthermore, future efforts should focus on strengthening integrated functional validation and mechanistic stratification across cellular, animal, and human models. Such research is essential to enhance our understanding of the pathogenicity and clinical significance of diverse *TBC1D4* variants. On this basis, it is imperative to promote genotype‐guided precision lifestyle interventions, stratified pharmacological management, and the development of specific molecular targeted therapies [46]. Such advancements will facilitate the clinical translation of *TBC1D4*‐related diseases from “genetic diagnosis” toward “precision therapeutics.”

## 7 Conclusion

In summary, this review systematically summarizes the molecular basis, pathogenic mechanisms, clinical features, diagnostic approaches, and therapeutic advances of monogenic diabetes caused by TBC1D4 mutations. Further research into this rare hereditary disorder of glucose metabolism will be important for improving clinical recognition, standardizing genetic testing, and guiding individualized interventions, thereby contributing to optimized diagnosis and long‐term management.

## Author Contributions

**Wenjing Wang:** conceived and designed the review, conducted the literature search, analyzed and interpreted the evidence, prepared the figures, and drafted the manuscript. **Peihan Gao:** contributed to literature retrieval, evidence synthesis, reference organization, and manuscript revision. **Lidan Ma** and **Bingzi Dong:** contributed to evidence analysis and interpretation and critically revised the manuscript for important intellectual content. **Xiaofang Sun:** contributed to the conception of the review and interpretation of the evidence, supervised the work, and critically revised the manuscript for important intellectual content. All authors contributed significantly to the work, reviewed and approved the final manuscript, and agree to be accountable for all aspects of the work in accordance with the ICMJE authorship criteria.

## Funding

This work was supported by the National Natural Science Foundation of China, Youth Project, No. 81800769.

## Disclosure

The authors have nothing to report.

## Ethics Statement

Ethics approval was not required for this review article because it is based exclusively on previously published literature and did not involve any new studies with human participants or animals conducted by the authors.

## Conflicts of Interest

The authors declare no conflicts of interest.

## Data Availability

Data sharing is not applicable to this article as no new datasets were generated or analyzed during the current study.
